# Immunofluorescence microscopy (IFM) analysis of primary ciliary dyskinesia (PCD) patients with suspected inner dynein arm defects (IDA)

**DOI:** 10.1186/2046-2530-1-S1-P23

**Published:** 2012-11-16

**Authors:** R Hjeij, NT Loges, A Becker-Heck, H Omran

**Affiliations:** 1 Universitätsklinikum Münster, Germany; 2 Department of Pediatrics, University Hospital Freiburg, Germany

## 

Primary ciliary dyskinesia (PCD), characterized by abnormal motility of cilia or flagella, is caused by defects of structural components such as inner dynein arms (IDAs). Recently high-speed videomicroscopy has substituted transmission electron microscopy (TEM) analysis as the “gold standard” for diagnosis. However, TEM is still the most widely used diagnostic tool in many countries. A recent study reported that isolated IDA defects is the most frequent (> 50%) ciliary defect in PCD, as detected by TEM (Theegarten, 2011). IFM analysis has shown in several studies that it can ascertain diagnosis such as in PCD variants caused by *DNAH5*, *DNAI1*, *DNAI2*, *KTU*, *LRRC50*, *CCDC39* and *CCDC40* mutations. IFM can provide a complete view of the ciliary axoneme and identify “partial” axonemal defects which may be misinterpreted by TEM (e.g. present in KTU mutant cilia). Here we performed IFM analysis of known PCD patients to identify the composition of dynein arm defects, including IDAs. Using antibodies specific to the IDA marker DNALI1, we determined that the frequency of isolated IDA defects is unexpected low (thus far, only 12 of ~800 PCD patients, ~1.5%). In contrast, we observe that IDA defects usually accompany DRC defects (absent/abnormal Gas11) or ODA defects (absent/abnormal DNAH5). Because the DNALI1 antibody is targeted against only 3 IDA isoforms out of 7, we are currently screening additional IDA-specific antibodies for IFM analysis to target the other IDA sub-types which escaped our analysis. However, our results suggest the percentage of isolated IDA defects might be lower than previously expected.

